# Effects of Gender Differences in *MTHFR* 677C > T and Homocysteine Level on the Occurrence of Adverse Pregnancy Outcomes

**DOI:** 10.1155/ijog/9133866

**Published:** 2025-08-22

**Authors:** Qian-nan Guo, Shi-xiu Liao, Hong-yan Liu, Yue Gao, Gui-yu Lou

**Affiliations:** ^1^Department of Medical Genetic Center of Henan Provincial People's Hospital (People's Hospital of Zhengzhou University), People's Hospital of Henan University, Zhengzhou, Henan, China; ^2^Henan Provincial Key Laboratory of Genetic Diseases and Functional Genomics, Zhengzhou, Henan, China

**Keywords:** biochemical pregnancy (BP), chromosome abnormality (CA), cleft lip and palate (CLP), homocysteine (Hcy), hyperhomocysteinemia (HHcy), methylenetetrahydrofolate reductase (MTHFR)

## Abstract

**Background:** The MTHFR 677C > T polymorphism in women has been associated with an increased risk of deep venous thrombosis and adverse pregnancy outcomes (APOs). However, research concerning its effects in men remains limited.

**Methods:** This study examined 662 adults with a history of pregnancies affected by chromosomal abnormalities (CAs: 343 females and 319 males), 137 adults with a history of pregnancies affected by cleft lip and palate (CLP: 71 females and 66 males), and 133 adults with a history of biochemical pregnancies (BPs: 65 females and 68 males), forming three case groups. A control group of 339 adults without APOs (221 females and 118 males) was studied. The genotypes of the *MTHFR* 677C > T polymorphism and Hcy levels were analyzed for all participants.

**Results:** Elevated Hcy levels were identified as a risk factor for CA, CLP, and BP in both adult females and males. The *MTHFR* 677C > T polymorphism was a risk factor for CA, CLP, and BP in females, whereas in males, it was a risk factor for CA and BP, but not for CLP. Individuals with the TT genotype exhibited the highest Hcy levels compared to those with CC and CT genotypes, across both genders and all groups. Males exhibited significantly higher Hcy levels and a significantly greater incidence of hyperhomocysteinemia compared to females across all groups.

**Conclusions:** The *MTHFR* 677C > T polymorphism was a gender-dependent risk factor for fetal CLP but was gender-independent for BP and fetal CA. Elevated Hcy levels were a gender-independent risk factor for BP, CLP, and CA. Individuals with the *MTHFR* 677TT genotype were more likely to have elevated Hcy levels, and there were notable gender differences in Hcy levels and hyperhomocysteinemia incidence.

## 1. Introduction

Adverse pregnancy outcomes encompass a range of atypical conditions that may arise during pregnancy, potentially affecting the health of either the mother or the fetus. These outcomes include, but are not limited to, spontaneous abortion, embryonic growth arrest, stillbirth, preterm birth, macrosomia, congenital anomalies, complications during childbirth, and neonatal mortality.

Methylenetetrahydrofolate reductase (MTHFR) is a crucial enzyme in the folate and homocysteine (Hcy) metabolic pathway, facilitating the conversion of 5,10-methylenetetrahydrofolate (5,10-MTHF) to 5-MTHF. 5-MTHF serves as a methyl donor to Hcy, resulting in the synthesis of methionine. Given the significance of folate and Hcy metabolism [1] in processes such as DNA and protein methylation, DNA and RNA synthesis and repair, neurotransmitter production, and ATP synthesis, it is hypothesized that Hcy, folate, and the MTHFR gene may influence adverse pregnancy outcomes.

In females, elevated Hcy levels have been associated with deep venous thrombosis [2] and several adverse pregnancy outcomes [3, 4], including recurrent pregnancy loss, preterm birth, and low birth weight in newborns. The *MTHFR* 677C > T polymorphism, one of the most prevalent single-nucleotide polymorphisms (SNPs) of the *MTHFR* gene, has also been identified as a risk factor for various adverse pregnancy outcomes, such as fetal aneuploidy [5], congenital heart defects (CHDs) [6], neural tube defects (NTDs) [7, 8], cleft lip and palate (CLP) [4, 9], and recurrent embryo loss [10]. In males, the *MTHFR* 677C > T polymorphism has been strongly associated with abnormal spermatogenesis [11] and infertility [11, 12]. However, the relationship between Hcy or the *MTHFR* 677C > T polymorphism and the occurrence of adverse pregnancy outcomes in male remains unclear.

Chromosomal abnormalities (CAs) and CLP in fetuses, along with biochemical pregnancy (BP) in females, represent three prevalent adverse pregnancy outcomes. In this study, we analyzed serum Hcy levels and the distribution of the *MTHFR* 677C > T polymorphism in adult females and males with a history of pregnancies affected by CA, CLP, and BP.

## 2. Methods

### 2.1. Subjects

All participants in this study were outpatients at the Medical Genetic Center of Henan Provincial People's Hospital. The cohort comprised 479 females and 453 males, all of whom experienced adverse pregnancy outcomes. Based on the specific types of adverse pregnancy outcomes, the cases were categorized into three groups: CA, CLP, and BP, each of which was confirmed through clinical and/or cytogenetic methods. The CA group included individuals with a history of pregnancies affected by aneuploidy, triploidy, or abnormal copy number variation (CNV) in the fetus. The CLP group consisted of individuals with a history of pregnancies affected by fetal CLP. The BP group included individuals with a history of BPs. All participants had a documented history of such adverse pregnancies prior to reaching the age of 35 and exhibited a normal peripheral blood karyotype.

In contrast, 339 subjects (comprising 221 females and 118 males) with a history of at least two healthy children and no adverse pregnancy outcomes at the time of recruitment or prior to the age of 35 were recruited as controls.

### 2.2. DNA Extraction

Genomic DNA was extracted from EDTA-anticoagulated peripheral blood utilizing the magnetic bead method, as provided by Xiamen Zeesan Biotech Co., Ltd.

### 2.3. Genotype Analysis

Genotype analysis was performed using polymerase chain reaction (PCR) and Sanger sequencing. The PCR primers, designed with Primer 5 software, target the MTHFR DNA sequence from GenBank: forward 5⁣′-GAA GCA GGG AGC TTT GAG GCT G-3⁣′, reverse 5⁣′-CCC ATG TCG GTG CAT GCC TTC-3⁣′. PCR conditions included an initial 5-min step at 95°C, followed by 33 cycles of 94°C 30 s, 57°C for 30 s, and 72°C for 37 s, with a final extension at 72°C for 9 min and a hold at 4°C.

### 2.4. Hcy Measurement

Fasting serum Hcy levels were measured using the ARCHITECT I2000SR, with participants abstaining from artificial B vitamin supplementation for at least 2 weeks prior to testing. In this study, fasting Hcy concentrations ≥ 15 *μ*mol/L were analyzed to assess hyperhomocysteinemia.

### 2.5. Statistical Analysis

SPSS Version 13.0 (Chicago, United States) was utilized for Chi-square (*χ*^2^) tests on allele/genotype distribution and odds ratio analysis. A two-tailed unpaired *T*-test was used to analyze age and Hcy concentration differences. The Chi-square goodness-of-fit test assessed Hardy–Weinberg equilibrium (HWE). Statistical significance was set at *p* < 0.05.

## 3. Results

There were no any significant differences (*p* > 0.05) in age distributions (Table 1) between each case group and the control group for either females or males. The genotype distributions (Tables 2 and 3) of the *MTHFR* 677C > T polymorphism were found to be in HWE (*p* > 0.05) across all case and control groups for both females and males.

Comparison between the case and control groups regarding *MTHFR* 677C > T distributions (Tables 2 and 3) reveals the following findings: (1) Within the CA group, a significant reduction in the frequencies of allele C and genotype CC was observed among both females and males. In contrast, there was a significant increase in the frequencies of allele T and genotype TT in females and a significant increase in the frequencies of allele T and genotype CT + TT in males. The presence of allele T was associated with a 2.562-fold and 1.678-fold increased risk of CA compared to allele C in females and males, respectively. Additionally, genotype TT was linked to a 5.460-fold and 2.761-fold increased risk of CA compared to genotype CC in females and males, respectively. Consequently, the *MTHFR* 677C > T polymorphism is identified as a gender-independent risk factor for the development of fetal CA. (2) In the CLP group, females exhibited a significant decrease in the frequencies of allele C and genotype CC, alongside a significant increase in the frequencies of allele T and genotype TT. The presence of allele T was associated with a 1.988-fold increased risk compared to allele C for the development of CLP in females, while genotype TT posed a 3.252-fold higher risk compared to genotype CC. Conversely, no significant changes were observed in the frequencies of alleles (C and T) or genotypes (CC, CT, and TT) among males. These findings suggest that the *MTHFR* 677C > T polymorphism serves as a gender-dependent risk factor for fetal CLP, predominantly affecting females. (3) In the BP group, the frequencies of allele C and genotype CC were significantly reduced in females, while the frequencies of allele T and genotype CT were significantly elevated. In males, the frequencies of allele C were significantly decreased, and the frequencies of allele T and genotype TT were significantly increased. The risk associated with allele T was 2.107 times higher in females and 1.705 times higher in males compared to allele C for the occurrence of BP. Additionally, genotype TT conferred a 4.863-fold and 2.490-fold increased risk compared to genotype CC in females and males, respectively. Thus, the *MTHFR* 677C > T polymorphism in both males and females was identified as a gender-independent risk factor for the development of BP. Overall, the *MTHFR* 677C > T polymorphism was determined to be a gender-dependent risk factor for fetal CLP, but a gender-independent risk factor for both BP and fetal CA.

A comparative analysis of Hcy concentration level between the case and control groups (Table 4) revealed that all case groups exhibited significantly elevated Hcy levels (*p* < 0.05) in both females and males. This finding suggests that increased Hcy levels constitute a risk factor for the development of CA, CLP, and BP in both genders, indicating that elevated Hcy levels serve as a gender-independent risk factor for BP, CLP, and CA. Furthermore, an examination of Hcy levels among subjects with the *MTHFR* 677CC genotype demonstrated that case subjects with the CC-genotype had significantly higher Hcy levels compared to control subjects with the same genotype, regardless of gender. This suggests that elevated Hcy levels may act as a gender-independent risk factor for the occurrence of CA, CLP, and BP, independent of the influence of the *MTHFR* 677C > T polymorphism on Hcy levels.

**Figure 1:**
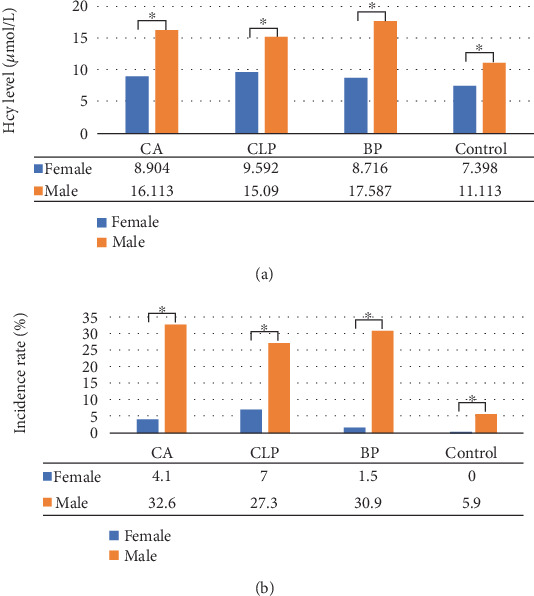
Differences in (a) Hcy levels and (b) hyperhomocysteinemia incidence between genders in the case and control groups. The data displayed in the accompanying table illustrate the mean Hcy levels (refer to Table 4 for details) and the average incidence rate of hyperhomocysteinemia (refer to Table 5 for details).⁣^∗^*p* values < 0.05. From left to right, *p* values were < 0.001 for all in (a), and < 0.001, < 0.002, < 0.001, and < 0.001 in (b).

Furthermore, we identified a substantial number of case subjects exhibiting hyperhomocysteinemia, with varying degrees of severity as detailed in Table 5. To investigate potential gender differences in the elevation of Hcy levels, we analyzed the distribution of Hcy levels and the incidence of hyperhomocysteinemia between females and males across all case and control groups. As illustrated in Figure 1, males exhibited significantly higher Hcy levels (*p* < 0.05) and a higher incidence rate of hyperhomocysteinemia (*p* < 0.05) compared to females in all groups. These findings indicate a gender difference in Hcy levels and the incidence of hyperhomocysteinemia.

**Figure 2:**
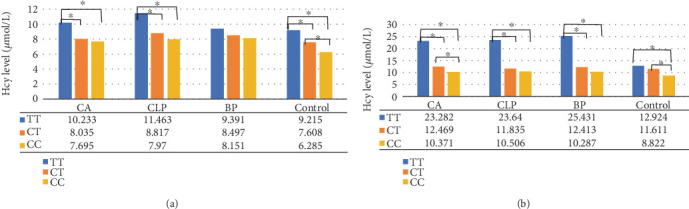
Comparison of Hcy levels among different genotyped subjects, stratified by sex: (a) females and (b) males. ⁣^∗^*p* value of less than 0.05, as determined by a two-tailed unpaired *t*-test, The data presented in the accompanying table represent the mean Hcy levels (refer to Table 4 for details). The *p* values for the comparisons, listed from left to right and top to bottom, are as follows: in panel (a), 0.003, < 0.001, 0.015, 0.026, < 0.001, < 0.001, and < 0.001 and in panel (b), < 0.001, 0.044, < 0.001, 0.002, < 0.001, 0.002 < 0.001, < 0.001, and < 0.001.

To further explore the influence of the *MTHFR* 677C > T polymorphism on Hcy levels and assess whether these impacts persist under pathogenic conditions, we conducted a comparative analysis of Hcy levels among subjects with different genotypes (CC, CT, and TT) in both the control and case groups. Our findings indicated a trend where individuals with the TT genotype exhibited the highest Hcy levels across all groups, regardless of sex, although statistical significance was not achieved in every group (Figure 2). In females (Figure 2a), the following observations were made: (1) Among CA females, those with the TT genotype exhibited an average Hcy level of 10.233 *μ*mol/L, which was significantly higher compared to those with the CT (8.035 *μ*mol/L) and CC (7.695 *μ*mol/L) genotypes. (2) Similarly, in CLP females, the TT genotype was associated with an average Hcy level of 11.463 *μ*mol/L, significantly exceeding the levels observed in CT (8.817 *μ*mol/L) and CC (7.970 *μ*mol/L) genotypes. (3) Among control females, the TT genotype corresponded to an average Hcy level of 9.215 *μ*mol/L, significantly higher than those of the CT (7.608 *μ*mol/L) and CC (6.285 *μ*mol/L) genotypes. (4) In BP females, although the Hcy levels did not differ significantly among the three genotypes, the TT genotype still exhibited the highest average Hcy level. These findings suggest that the *MTHFR* 677C > T polymorphism influences the elevation of Hcy levels in females under both healthy and pathological conditions, with the 677TT genotype exerting the most pronounced effect.

In males (Figure 2b), individuals with the TT genotype exhibited the highest average Hcy levels across all groups: (1) The TT-genotyped CA males had an average Hcy level of 23.282 *μ*mol/L, which was significantly elevated compared to CT (12.469 *μ*mol/L) and CC (10.371 *μ*mol/L) genotyped CA males. (2) TT-genotyped CLP males had an average Hcy level of 23.640 *μ*mol/L, significantly surpassing the Hcy levels of CT (11.835 *μ*mol/L) and CC (10.506 *μ*mol/L) genotyped CLP males. (3) TT-genotyped BP males demonstrated an average Hcy level of 25.431 *μ*mol/L, significantly higher than those of CT (12.413 *μ*mol/L) and CC (10.287 *μ*mol/L) genotyped BP males. (4) Among control males, those with the TT genotype had an average Hcy level of 12.924 *μ*mol/L, significantly higher than the CC-genotyped control males (8.822 *μ*mol/L). These findings indicate that the *MTHFR* 677C > T polymorphism contributes to elevated Hcy levels in males under both healthy and pathological conditions, with the 677TT genotype exerting the most pronounced effect. In summary, the *MTHFR* 677C > T polymorphism was found to influence the elevation of Hcy levels in both females and males, under both healthy and pathological conditions, with the 677TT genotype exerting the most pronounced effect on increasing Hcy levels.

## 4. Discussion

In this study, males generally exhibited significantly higher Hcy levels and a higher incidence of hyperhomocysteinemia compared to females across all the case and control groups. This finding suggests a gender difference in Hcy levels, with males being more susceptible to developing hyperhomocysteinemia than females. Elevated Hcy levels were identified as a gender-independent risk factor for the occurrence of CA, CLP, and BP in this study. Numerous other studies have reported an association between increased Hcy levels and an elevated risk of various diseases [13, 14], particularly in adult females [15–18]: elevated Hcy levels have been identified as risk factors for recurrent early abortion or miscarriage, pregnancy-induced hypertension, fetal Down syndrome, fetal CHDs, and NTDs. Furthermore, the administration of 30 *μ*M Hcy into the neural tube lumen [19] or 0.5 mM HTL (active metabolite of Hcy) into the neural groove [16, 20] has been demonstrated to induce heart defects or NTDs in chicken embryos, respectively. In mouse models of NTD, elevated levels of Hcy have been observed to impact epigenetic mechanisms by disrupting histone methylation at various lysine residues on histone H3, leading to dysregulation of gene expression [16, 21, 22]. Specific histone H3 methylations, such as H3K27me3, H3K36me3, and H3K79me2, play critical roles in stage-specific gene expression during embryonic development and in the temporal regulation of programmed histone modifications [21, 23]. Consequently, elevated Hcy levels appear to be detrimental not only to individuals but also to their progeny by influencing epigenetic mechanisms. Moreover, elevated Hcy levels can disrupt mitochondrial respiratory chain metabolism, which may subsequently affect chromosome segregation [24]. In this study, elevated Hcy levels in both females and males were identified as risk factors for the occurrence of CA,CLP and BP. Therefore, the impact of Hcy on mitochondrion activity or the epigenetic mechanisms of germline cells may contribute to the development of CA, CLP, and BP.

In this study, the *MTHFR* 677C > T polymorphism was identified as a gender-independent risk factor for the development of CA and BP. However, it was identified as a risk factor for the occurrence of CLP exclusively in females, with no significant association observed in males. These findings regarding the *MTHFR* 677C > T polymorphism differ from the effects of elevated Hcy levels, which were identified as a gender-independent risk factor for the occurrence of all these conditions in both females and males. Considering that males can influence embryonic development solely through the genetic information contained in their sperm cells, the impact of the male *MTHFR* 677C > T polymorphism in sperm may not be associated with the formation of fetal CLP. The development of CLP in the fetus may be exclusively affected by maternal factors.

In adult human females, the *MTHFR* 677C > T polymorphism has been identified as a risk factor not only for the occurrence of fetal NTD, CHD, and CLP [4, 9, 25] but also for fetal nonmosaic aneuploidy [26]. This suggests that the *MTHFR* 677C > T polymorphism in females likely influences germline cell division and fetal development, potentially leading to CA in fetus or embryo, which may result in miscarriage or congenital defects. In adult human males, the *MTHFR* 677C > T polymorphism, particularly the 677TT genotype has been strongly associated with abnormal spermatogenesis [11], infertility, hypomethylation of sperm DNA [27], and embryonic heart defects [5, 28]. These findings in males indicate that the *MTHFR* 677C > T polymorphism is also likely to impact germline cell division and fetal development.

Moreover, the animal model provided compelling evidence that the *MTHFR* gene plays a critical role in germline cell development and the occurrence of birth defects. In *mthfr*-deficient mouse models, mice displayed the following: (1) impaired growth, delayed development, and increased mortality and morbidity [29, 30], which parallel adverse pregnancy outcomes observed in humans; (2) hyperhomocysteinemia [29, 31]; (3) hypomethylation of sperm DNA [32], along with abnormal spermatogenesis and infertility [30]; (4) *mthfr* was highly expressed in male reproductive tissues [33]; and (5) in a transgenic mouse model with a point mutation analogous to the human *MTHFR* 677C > T variation [34], the TT-genotyped male mice exhibited sperm DNA hypomethylation similar to that observed in humans [35]. Collectively, studies in both mice and humans suggest that the *MTHFR* gene of both females and males plays a significant role in the development of germline cells, potentially through its influence on DNA methylation and the epigenetic mechanisms governing germline cells.

In this study, TT-genotyped subjects exhibited the highest average Hcy levels compared to those with CC and CT genotypes, across both the case and control groups. This observation aligns with existing knowledge that the TT genotype significantly reduces MTHFR activity to approximately 30% of the activity observed in the CC wild-type genotype, which is considerably lower than the heterozygous CT variant, which maintains about 65% of the CC wild-type MTHFR activity [36]. Consequently, the MTHFR 677C > T polymorphism influences the elevation of Hcy levels, with the TT genotype being significantly associated with higher Hcy levels compared to the CT and CC genotypes [36, 37].

Furthermore, this study found that all CC wild-type subjects, regardless of sex, across the three case groups exhibited significantly higher Hcy levels compared to their CC genotype counterparts in the control group. This finding suggests the presence of additional factors influencing Hcy levels beyond the MTHFR 677C > T polymorphism alone, and elevated Hcy levels may act as an independent risk factor for the development of CA, CLP, and BP, irrespective of the influence of the *MTHFR* 677C > T polymorphism on Hcy levels.

In both human and transgenic mouse models, Hcy levels consistently exhibit an inverse correlation with folate levels. Folate supplementation in women [38–43] during the preconception and gestational periods has been shown to effectively prevent various birth defects, such as fetal CHD, NTDs, and low birth weight. This beneficial effect is similarly observed in maternal *MTHFR* deficient (*MTHFR* −/−) mice [44]. Additionally, folate supplementation in men has been shown to positively influence spermatogenesis [35, 45]. Research conducted by Naz et al. [46] suggests that folate treatment can partially rectify germline methylation errors in adult male rats. Consequently, folate supplementation in both adult men and women may be effective in preventing Hcy or *MTHFR* 677C > T related APOs, such as CA, CLP, and BP observed in this study. Therefore, further research on the efficacy of folate supplementation in the prevention of CA, CLP, and BP is warranted.

## 5. Conclusion

In this study, the *MTHFR* 677C > T polymorphism was identified as a gender dependent risk factor for fetal CLP, while it served as a gender-independent risk factor for BP and fetal CA. Elevated Hcy levels were determined to be a gender-independent risk factor for BP, CLP, and CA. Subjects with the *MTHFR* 677TT genotype exhibited a higher likelihood of elevated Hcy levels compared to those with the other two genotypes (CT and CC). Notable gender differences were observed in Hcy levels and the prevalence of hyperhomocysteinemia. Overall, the *MTHFR* 677C > T polymorphism and elevated Hcy levels in both males and females were found to influence the incidence of adverse pregnancy outcomes.

## Figures and Tables

**Table 1:** Age (years) distributions.

**Groups**	**Gender**	**MTHFR 677C > T**			**Total**
		**CC**	**CT**	**TT**	
CA	Female (343)	30.630 ± 3.504	29.655 ± 3.932	29.389 ± 3.990	29.697 ± 3.905
	Male (319)	31.612 ± 3.978	30.458 ± 4.580	30.897 ± 3.988	30.790 ± 4.267
CLP	Female (71)	30.588 ± 4.938	29.296 ± 3.123	28.889 ± 4.136	29.493 ± 4.126
	Male (66)	30.438 ± 4.704	30.700 ± 3.687	30.650 ± 4.368	30.621 ± 4.094
BP	Female (65)	29.750 ± 3.327	30.184 ± 3.826	31.158 ± 5.178	30.415 ± 4.179
	Male (68)	29.539 ± 5.206	32.222 ± 4.397	31.071 ± 5.436	31.235 ± 5.023
Control	Female (221)	30.663 ± 2.677	30.161 ± 2.551	29.238 ± 2.835	30.181 ± 2.692
	Male (118)	31.892 ± 3.806	30.918 ± 3.451	31.906 ± 6.275	31.492 ± 4.474

*Note:* Data were presented as “mean ± standard deviation”.

**Table 2:** Analysis of the distribution and odds ratios for the *MTHFR* 677C > T polymorphism in females.

**Allele or genotype**	**CA (%)**	**CLP (%)**	**BP (%)**	**Control (%)**
C	253⁣^∗^ (36.9)	61⁣^∗^ (43.0)	54⁣^∗^ (41.5)	265 (60.0)
T	433⁣^∗^ (63.1)	81⁣^∗^ (57.0)	76⁣^∗^ (58.5)	177 (40.0)
CC	54⁣^∗^ (15.7)	17⁣^∗^ (23.9)	8⁣^∗^ (12.3)	86 (38.9)
CT	145 (42.3)	27 (38.0)	38⁣^∗^ (58.5)	93 (42.1)
TT	144⁣^∗^ (42.0)	27⁣^∗^ (38.0)	19 (29.2)	42 (19.0)
Subjects	343	71	65	221
*P* for H-W	0.088	0.059	0.101	0.066
T vs. C	OR: 2.562; 95% CI: 2.005~3.275	OR: 1.988; 95% CI:1.356~2.915	OR: 2.107; 95% CI:1.416~3.135	—
CT vs. CC	OR: 2.483; 95% CI:1.618~3.811	OR: 1.469; 95% CI:0.749~2.882	OR: 4.392; 95% CI:1.941~9.942	—
TT vs. CC	OR: 5.460; 95% CI:3.366~8.857	OR: 3.252; 95% CI:1.599~6.616	OR: 4.863; 95% CI:1.968~12.018	—

Abbreviation: H-W, Hardy–Weinberg equilibrium.

⁣^∗^Chi-square analysis *p* value of less than 0.05 between the case and control groups.

**Table 3:** Analysis of the distribution and odds ratios for the *MTHFR* 677C > T polymorphism in males.

**Allele or genotype**	**CA (%)**	**CLP (%)**	**BP (%)**	**Control (%)**
C	251⁣^∗^ (39.3)	62 (47.0)	53⁣^∗^ (39.0)	123 (52.1)
T	387⁣^∗^ (60.7)	70 (53.0)	83⁣^∗^ (61.0)	113 (47.9)
CC	49⁣^∗^ (15.4)	16 (24.2)	13 (19.1)	37 (31.4)
CT	153 (48.0)	30 (45.5)	27 (39.7)	49 (41.5)
TT	117 (36.7)	20 (30.3)	28⁣^∗^ (41.2)	32 (27.1)
Subjects	319	66	68	118
*P* for H-W	0.930	0.477	0.173	0.068
CT + TT	270⁣^∗^ (84.6)	50 (75.8)	55 (80.9)	81 (68.6)
T vs C	OR: 1.678; 95% CI:1.242~2.267	OR: 1.229; 95% CI:0.802~1.883	OR: 1.705; 95% CI:1.110~2.618	—
CT vs CC	OR: 2.358; 95% CI:1.382~4.024	OR: 1.416; 95% CI:0.674~2.973	OR: 1.568; 95% CI:0.714~3.447	—
TT vs CC	OR: 2.761; 95% CI:1.548~4.925	OR: 1.445; 95% CI:0.643~3.249	OR: 2.490; 95% CI:1.108~5.600	—

Abbreviation: H-W, Hardy–Weinberg equilibrium.

⁣^∗^Chi-square analysis *p* value of less than 0.05 between the case and control groups.

**Table 4:** Hcy (*μ*mol/L) distributions in the case vs. control groups.

**Hcy levels**	**Females**				**Males**			
	**CA (343)**	**CLP (71)**	**BP (65)**	**Control (221)**	**CA (319)**	**CLP (66)**	**BP (68)**	**Control (118)**
CC genotyped subjects	7.695 ± 1.831 (*p* < 0.001)	7.97 ± 2.327 (*p* < 0.001)	8.151 ± 1.520 (*p* < 0.001)	6.285 ± 1.293	10.371 ± 2.763 (*p* = 0.005)	10.506 ± 2.181 (*p* = 0.005)	10.287 ± 2.364 (*p* = 0.025)	8.882 ± 1.697
Total subjects	8.904 ± 5.061 (*p* < 0.001)	9.592 ± 4.106 (*p* < 0.001)	8.716 ± 2.216 (*p* < 0.001)	7.398 ± 2.102	16.112 ± 10.649 (*p* < 0.001)	15.09 ± 10.672 (*p* < 0.001)	17.587 ± 13.088 (*p* < 0.001)	11.113 ± 3.485

*Note:* Data is presented as “average ± standard deviation”.

**Table 5:** Number of subjects with hyperhomocysteinemia (Hcy ≥ 15 *μ*mol/L).

**HHcy**	**CA**		**CLP**		**BP**		**Control**	
	**Female (343)**	**Male (319)**	**Female (71)**	**Male (66)**	**Female (65)**	**Male (68)**	**Female (221)**	**Male (111)**
15≦HCY≦20	8	41	4	8	1	5	0	4
20 < HCY≦30	3	30	0	5	0	7	0	1
30 < HCY≦40	0	16	1	1	0	1	0	1
40 < HCY≦50	1	5	0	1	0	5	0	0
HCY > 50	2	12	0	3	0	3	0	0
Total HHcy	14 (4.1%)	104 (32.6%)	5 (7.0%)	18 (27.3%)	1 (1.5%)	21 (30.9%)	0 (0.0%)	7 (5.9%)

## Data Availability

All relevant data are within the manuscript.
